# Is there still yaws in Nigeria? Active case search in endemic areas of southern Nigeria

**DOI:** 10.1371/journal.pntd.0011753

**Published:** 2023-11-20

**Authors:** Ngozi Ekeke, Francis S. Iyama, Joseph N. Chukwu, Kingsley Asiedu, Michael Marks, Babatunde Omotowo, Olanike Agwu-Umahi, Victor O. Nvene, Shiloh Paul, Charles C. Nwafor, Anthony O. Meka, Chinwe C. Eze, Okechukwu E. Ezeakile, Martin I. Njoku, Ngozi N. Murphy-Okpala

**Affiliations:** 1 German Leprosy and Tuberculosis Relief Association (GLRA), Enugu, Nigeria; 2 Department of Control of Neglected Tropical Diseases, WHO, Geneva, Switzerland; 3 Department of Clinical Research, London School of Hygiene and Tropical Medicine (LSHTM), London, England; 4 Institute of Public Health, University of Nigeria, Nsukka, Nigeria; 5 Neglected Tropical Diseases Division, Federal Ministry of Health, Abuja, Nigeria; University of California Davis School of Veterinary Medicine, UNITED STATES

## Abstract

**Background:**

Yaws is a disease caused by the bacteria *Treponema pallidum* subspecies *pertenue*, which is most commonly seen among children below 15 years. In the twentieth century yaws was endemic in Nigeria but eradication strategies markedly reduced the disease burden. Currently there is minimal data on the ongoing transmission of yaws in Nigeria, despite reports of confirmed yaws cases in neighbouring West African countries.

**Methods:**

We conducted both community and school-based active yaws case search among school-aged children in southeast Nigeria. Children were screened by trained community volunteers. Suspected yaws cases were clinically reviewed and tested using rapid diagnostic serological tests.

**Results:**

Between February and May 2021, up to 28 trained community volunteers screened a total of 105,015 school children for yaws. Overall, 7,706 children with various skin lesions were identified. Eight (8) suspected cases of yaws were reported, reviewed and screened, but none was confirmed using rapid diagnostic tests. The four most common skin conditions identified were scabies (39%), papular urticaria (29%), tinea corporis (14%) and tinea capitis (12%).

**Conclusions:**

No case of yaws was confirmed in this large population of children in south-east Nigeria. Continuous community awareness and yaws case finding activities have been recommended across Nigeria.

## Author summary

In our research, we searched for a disease called yaws, which is caused by a type of bacteria. Yaws mostly affects children under 15 years old. In the past, yaws was a big problem in Nigeria, but efforts to get rid of the disease have been successful in reducing the number of cases.

However, we still needed to check if yaws is still spreading in Nigeria. Even though other nearby countries reported cases of yaws, there wasn’t much information about what was happening in Nigeria. So, we did a study in southeast Nigeria. We looked for yaws in school-aged children by visiting communities and schools. If a child had skin problems that looked like yaws, we checked them carefully and used quick tests to see if they had yaws.

After checking about 105,015 schoolchildren, we found 7,706 children with skin issues, but when we tested them for yaws, none of them actually had the disease.

The most common skin problems we found were scabies, a kind of rash (papular urticaria), and some fungal skin infections.

We suggest that Nigeria should keep looking for cases, just to be sure the disease doesn’t come back.

## Introduction

In the mid twentieth century, Nigeria was known to be highly endemic for yaws [1–3]. Yaws was reported to have occurred in all five provinces of the previous Eastern Region of Nigeria, with the old Nsukka Division reporting a particularly high burden of disease [2]. A 1953 survey in Nsukka Division screened 95% of the resident population and found the prevalence of infectious yaws varied from 1.5% to 11.5% between communities. Subsequently, 12,221 infectious cases, 42,553 late cases, and 328,995 latent cases and contacts were treated using community wide mass treatment with penicillin. In subsequent resurveys the prevalence was found to have fallen to almost zero [2]. In a study conducted by Nnoruka yaws was not identified among any of the 2,871 patients at the dermatology clinic of the University of Nigeria Teaching Hospital in Enugu [4]. Despite the official position that yaws no longer existed in Nigeria, a study on filariasis in the Hawal River valley in northeastern Nigeria reported incidental findings of yaws in 1998 [5]. Interest in yaws eradication has been revitalized since 2012. However, one of the main challenges for the current eradication efforts has been identified to be incomplete information on the true burden and spatial distribution of the disease [6]. The disease is still known to be endemic in at least 15 countries predominantly in West Africa, South East Asia and the Pacific [6–8]. In the WHO African region, seven countries reported cases of yaws to the World Health Organization (WHO) including several that are in close proximity to, or border Nigeria.

Nigeria currently has insufficient data on the occurrence of yaws, even though there is robust evidence of yaws transmission in neighboring countries. It is not yet clear if the current status of yaws in Nigeria implies the absence of evidence or the evidence of its absence. We therefore sought to conduct active case search for yaws in previously endemic districts in Nigeria.

## Methodology

### Ethical approval

This study was reviewed and approved by the Health Research Ethics Committee of the University of Nigeria Teaching Hospital (UNTH) Enugu with certificate number: HREC/05/01/2008B-FWA00002458-1RB00002323. In addition, official permission to conduct this survey was duly obtained from the Enugu State Ministry of Education, State Ministry of Health, Enugu State Primary and Post Primary School Management Boards, Heads of selected schools, Parents Teachers Associations of selected schools and community leaders. Verbal consent of relevant school management authorities, parents and guardians were obtained in the study locations. Information obtained was used strictly for the purpose of the research and not in any publication or report.

**Fig 1:**
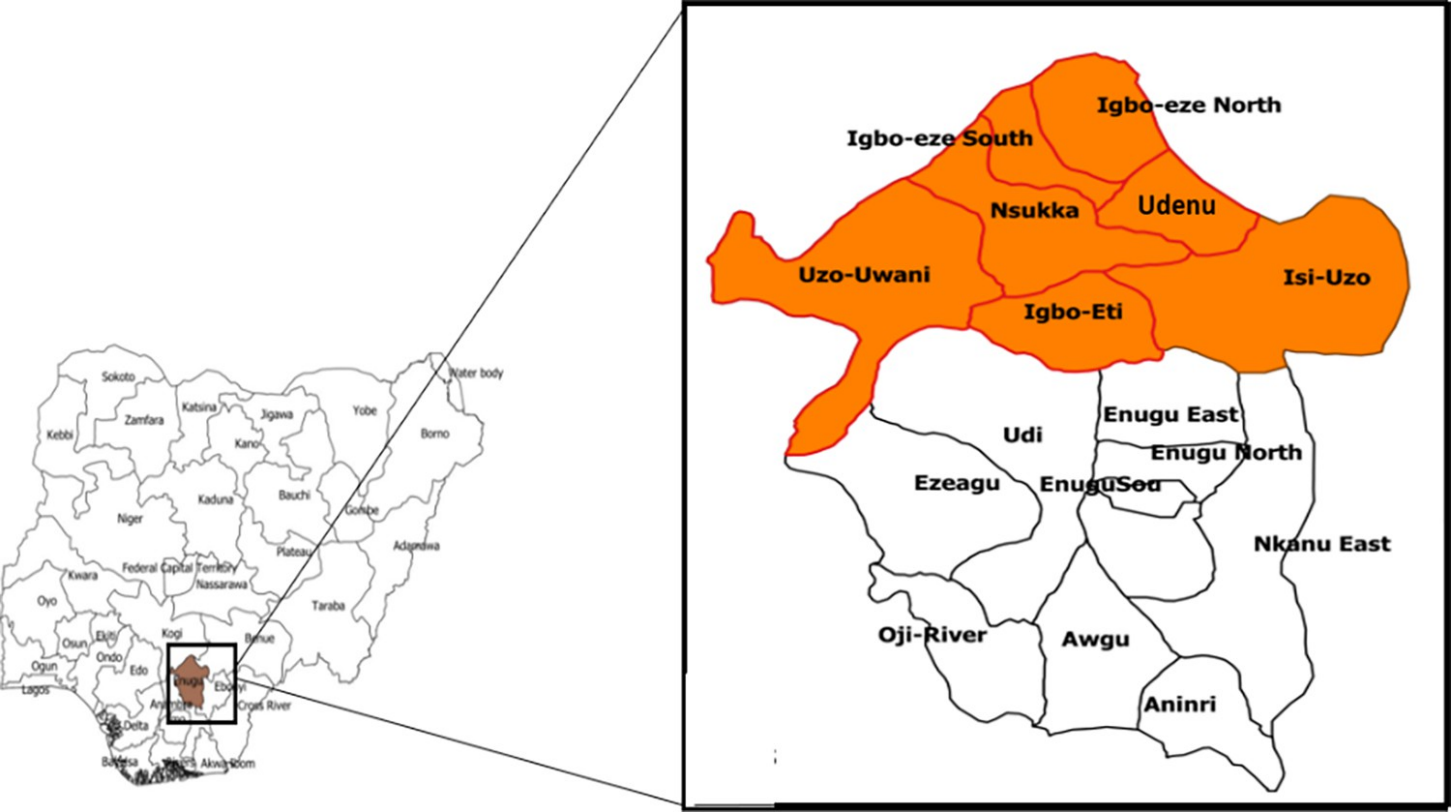
Map of Nigeria showing Enugu State and the study area (LGAs in old Nsukka Division). Direct link to the base layer of the map: https://gadm.org/download_country.html Link to the terms of use / license information for the base layer image or shapefile: https://gadm.org/license.html.

We conducted a series of community and school-based cross-sectional surveys among children in the old Nsukka division of Enugu State, southeast Nigeria. In this study, the selection of 61 out of 113 wards across the 7 LGAs was based on remote hard-to-reach locations identified by LGA health authorities in the study area during prior advocacy visits. For the wards that were not selected for the survey, care was taken to carry out intensive community awareness/ campaigns on yaws using posters, flyers and booklets provided by the WHO. Contact numbers of our field workers were made available to the community members to refer suspected cases. Moreover, there was no major events that could have necessitated mass movement of school children within the study period.The survey took place between February and May 2021 Fortunately, there were no major events requiring mass movement of people during the study period. In each Local Government Area (LGA) equivalent to a district, two community volunteers (motivated, young, unemployed university graduates) were recruited and trained to conduct this survey among school-age children in all the seven LGAs known to have previously reported high cases of yaws. These areas now make up the old Nsukka Division (Fig 1).

### Study location

This study location was purposively selected as it had been previously reported to be hyper- endemic for yaws, with mass eradication campaigns carried out there in the 1950s [9]. Old Nsukka Division is presently made up of seven Local Government Areas. They are Nsukka, Igbo-Etiti, Igboeze North, Igboeze South, Uzo-Uwani, Udenu and Isi-Uzo LGAs. [10]. Based on the 2006 national census the total population of the 7 LGAs is estimated to be 2,084,966 [11]. Based on the registry of schools there are 537 public primary schools, 121 public secondary schools and 732 private schools.

Since majority of active yaws occurs in children under 15 years of age [7], study population included all children (both in-school and out-of-school) from all seven LGAs, including children with disability. Inclusion criterion was children aged 2-15years.

As it is not possible to power a study to detect the complete absence of disease we instead powered the study to detect a very low prevalence of active yaws. To detect a prevalence of 0.68% (estimated from a survey in Ghana [12]) with a precision of 0.34 and a design effect of 6 we calculated that we would need to enroll approximately 13,466 children. We considered this a minimum sample size and aimed to enroll a larger sample given the highly clustered nature of yaws [13].

We used multi-stage sampling to recruit participants for the study. In the seven selected LGAs we used purposive sampling for selection of remote, rural, hard-to-reach sub-districts. These were identified by district health workers, in particular the Local Government TB and Leprosy Supervisors as well as the Local Immunization Officers. Finally, we selected all schools from these identified wards.

In each selected ward, community awareness on yaws was carried out in all the neighboring communities around each selected school. This was done in collaboration with the head teachers of each school in the area. These campaigns and screening for yaws were carried out both in schools and in congregate settings such as religious groups, markets and other social gatherings in the community. Care was taken to ensure out-of-school children in the communities were reached by the field workers (community volunteers). With each school and its community forming a cluster all the students in each cluster were studied.

Twenty-eight (28) volunteer field workers were recruited to conduct the active yaws search. The researchers had the opportunity to receive capacity building (in initial training the trainers’ workshop) facilitated by yaws expert from the World Health Organization and the London School of Hygiene and Tropical Medicine. The training focused on examination of skin lesions to differentiate yaws from other skin diseases as well as laboratory testing procedure using rapid diagnostic tests kits. The facilitators provided training documents which were adapted for use in training of field workers. Staff of the German Leprosy and TB Relief Association (GLRA) Nigeria and those of Institute of Public Health, University of Nigeria, Nsukka then conducted a two-day residential training for the field workers (community volunteers). In addition to training on recognition of yaws and use of rapid diagnostic testing, the field workers were mentored on the use of mobile applications for real time data collection and information sharing using the Open Data Kit (ODK) for data reporting.

**Fig 2:**
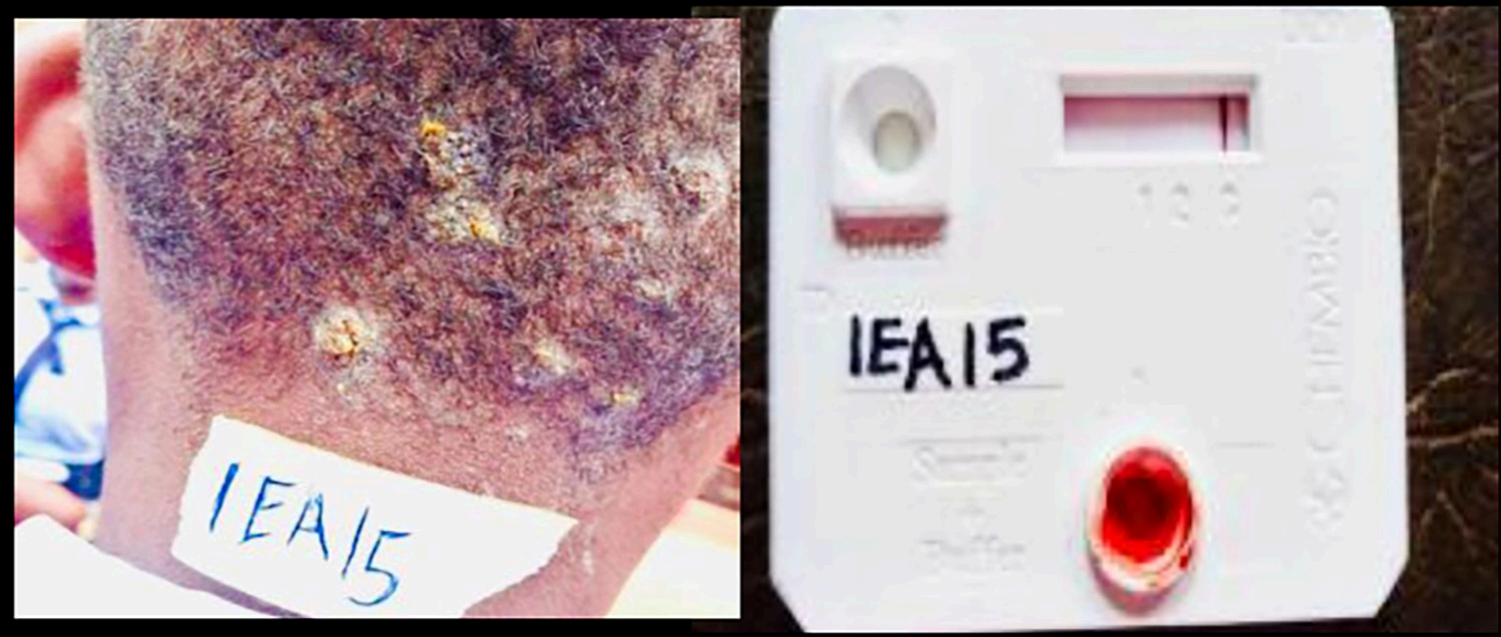
Clinical presentation and DPP negative result of a 14 year old male.

**Fig 3:**
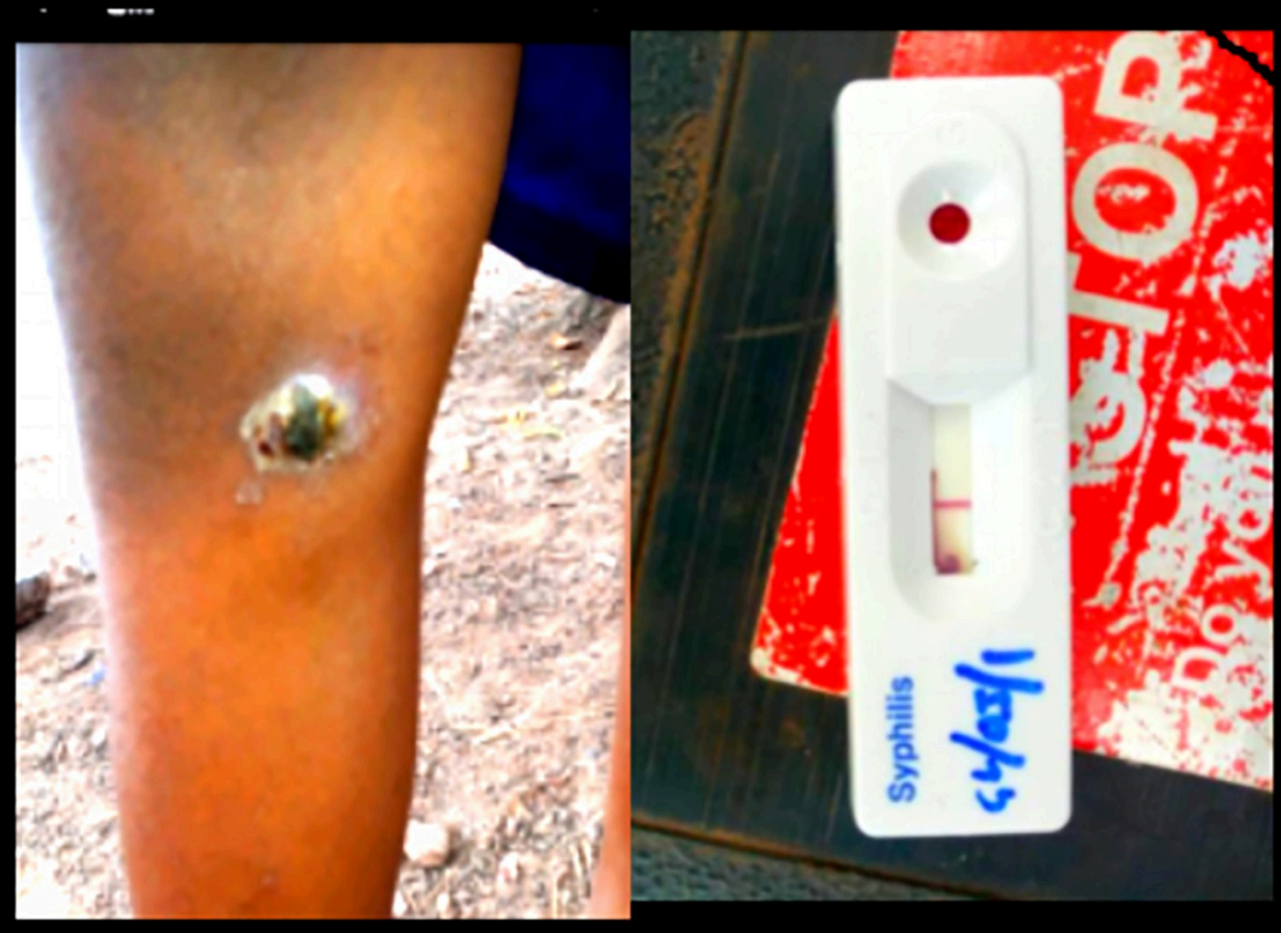
Clinical presentation and SD Bioline negative result of an 11 year old female.

**Fig 4:**
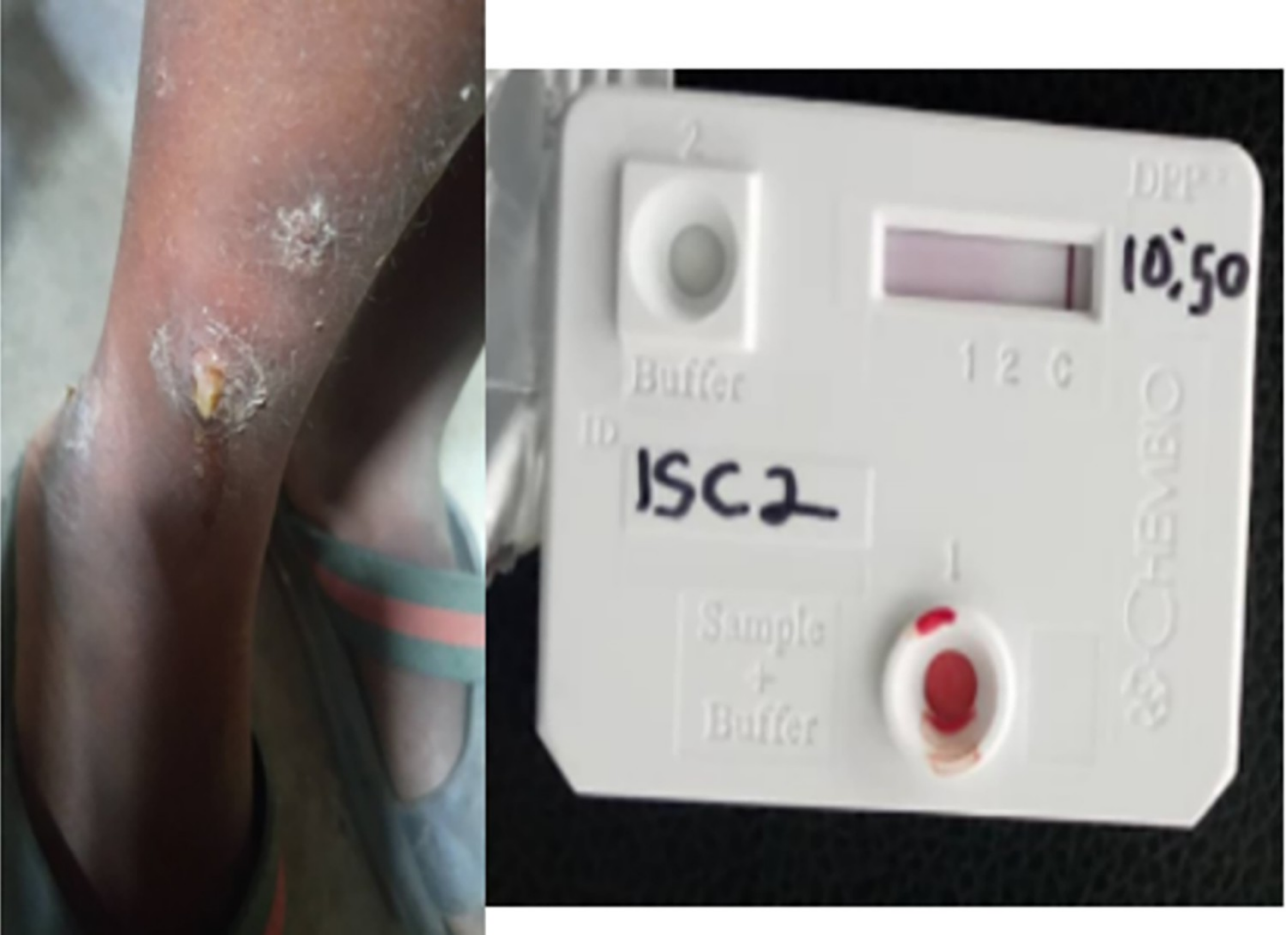
Clinical presentation and DPP negative result of a 6 year old female.

Two field workers were assigned to each of the seven LGAs for adequate coverage. Each team of field workers visited selected schools and communities in their assigned LGAs for sensitization and screening. A minimum of two schools and one community were visited every week by each trained community volunteer. Photographs of suspected skin lesions were taken and shared with the research team who provided feedback accordingly. Essential medicines for common skin conditions such as fungal infections and scabies were provided to accompany each field visit. Suspected skin lesions (for yaws) were referred for skin examination and testing on an agreed date and at designated centres (preferably a school or nearby health facility). Research team visited in person to examine skin lesions suspected for yaws. (Figs 2, 3 and 4) take appropriate photographs, conducted rapid diagnostic tests and prepared to send any positive samples for PCR confirmation.

Two rapid diagnostic tests (RDTs), namely the DPP (Dual Path Platform) and SD Bioline 3.0, were employed in this study for the detection of yaws. These RDTs are designed to provide quick and reliable results for the presence of yaws infection in individuals. The DPP test was manufactured by Chembio Diagnostic Systems, Inc, Medford, New York, while the SD Bioline 3.0 test was manufactured by Standard Diagnostics Korea Co., Ltd Gyeonggi-do, Republic of Korea. Both tests are well-established and widely used for the detection of yaws in various epidemiological settings [13].

The SD BIOLINE Syphilis 3.0 is a solid phase immunochromatographic assay for the qualitative detection of antibodies of all isotypes (IgG, IgM, IgA) against Treponema pallidum (TP) in human serum, plasma or whole blood.

It contains a membrane strip, which is pre- coated with recombinant Treponema pallidum antigens (17,15KDa) on test band region. The recombinant Treponema pallidum antigens-colloid gold conjugate (17,15KDa), patient sample and sample diluent moves along the membrane chromatographically to the test region (T) and forms a visible line as the antigen-antibody-antigen gold particle complex forms. Therefore, the formation of a visible line in the test region (T) indicates a positive result for the detection of Treponema pallidum specific antibodies (IgG, IgA, IgM). When the Treponema pallidum specific antibodies (IgG, IgA, IgM) are absent in the sample, no visible color band in the test region (T) [14].

The Chembio DPP Syphilis Screen & Confirm Assay employs a unique combination of protein A and anti-human IgM antibody, which are conjugated to colloidal gold particles. It also utilizes a recombinant antigen of T. pallidum and synthetic antigens for non-Trepo- nema, separately bound to the membrane solid phase. The sample is applied to the Well 1 along with the Running Buffer. Five minutes after adding the sample + Running Buffer to Well 1, additional Running Buffer is added to BUFFER Well 2. The Running Buffer allows gold conjugates to migrate and bind to the antibody/antigen complex at the test lines. If the antibodies to syphilis (Treponemal and non-Treponemal) are present in the sample then two pink/purple lines (1 and 2) are produced. In the absence of anti- Treponemal and anti-non-Treponemal antibodies, there are no pink/purple lines in the TEST (1 and 2) area. Unbound conjugated gold particles continue to migrate along the membrane and produce a pink/purple line in the CONTROL (C) area [15].

Digital technology, especially ODK and WhatsApp were deployed to monitor project implementation and support field workers to ensure quality delivery. A dedicated Whatsapp group was created for effective and efficient communication between the field workers (community volunteers) and the research (technical) team.

## Results

A total of 105,015 school-age children were screened in 809 schools and 790 communities from the 7 Local Government Areas. Overall 52.4% of children screened were female. A total of 7,706 (7.3%) referrals were made by community volunteers. After review of the referral information by the research team 8 participants, 2 male and 6 female, with suspected yaws were identified. (Figs 2, 3 and 4) On review by the research team of German Leprosy and Tuberculosis Relief Association (GLRA), it was observed that majority of the skin lesions were diagnosed as scabies (39%), papular urticaria (29%) and dermatophyte infections (26%) and others 6% (Table 1). Incidentally, a case of tuberculosis and that of leprosy were also identified and referred appropriately for treatment.

**Table 1:** Categories of skin lesions identified during active yaws case search.

Diagnosis	Frequency	Percentage
Yaws	0	0%
Scabies	3,005	39%
Papular urticaria	2,235	29%
Tinea corporis	1,079	14%
Tinea capitis	925	12%
Others (infected traumatic ulcers, etc)	462	6%
TOTAL	7,706	100%

## Discussion

In this study mass screening of school-age children in selected hard-to-reach communities in old Nsukka Division was conducted to ascertain the presence or otherwise of yaws in the population. None of the suspected yaws cases identified from the schools and communities were confirmed as Yaws on serological testing (Table 2). To our knowledge this is the first study conducted to assess for any occurrence of yaws in this region since presumed yaws elimination.

**Table 2:** Report of skin conditions identified during yaws screening in the LGAs.

LGAs	Total number screened	Total number with skin lesions	Total Tested	Total positive: SD- Bioline and DPP
Igbo Eze South	31507	2074	3	0
Igbo-Etiti	16857	1232	3	0
Igbo Eze North	15810	592	1	0
Nsukka	15479	579	0	0
Isi-Uzo	9870	1885	1	0
Uzo-Uwani	9815	925	0	0
Udenu	5677	419	0	0
TOTAL	105,015	7,706	8	0

In our study we screened a high number of school-age children both in schools and communities, especially in the selected locations identified by local health authorities as hard-to-reach communities/wards.

We observed 7.3% skin disease prevalence among school age children which is lower than 35.3% reported in three semi urban communities in Obio/Akpor Local Government Area of Rivers State [16] and also below the 35% to72.3% reported in Nigeria [17–19]. The remarkably low skin disease prevalence in this study could be a reflection of the high hygiene awareness and practices during the emerging post-COVID era when the study was carried out. Despite the fact that no yaws cases were confirmed in this region, we identified other skin diseases among the children. Scabies, papular urticaria and fungal skin infections were the most common skin conditions observed. These findings are similar to a study done in Obio/Akpor Local Government Area of Rivers State where dermatophyte infections,papular urticaria and scabies where all common [16]. These skin diseases were also common among school children in Côte d’Ivoire [20].

The successful community entry and acceptance was a result of robust advocacy visits to relevant stakeholders including community gate-keepers. Free provision of essential medicines for treatment of other skin infections identified during field work also promoted acceptance.We also found that widespread access to internet and mobile phones facilitated tele-dermatology and that this was able to compensate for the lack of experts in the field [20]. However, technology comes with its downsides, whereas photographs of skin lesions were transmitted to researchers for real-time review and decision making, some of the pictures shared from the field by the field workers were of poor quality and therefore could not be reviewed.

Only 8 out of the 7706 skin lesions presented were suspected to be yaws and therefore were tested. This low number may be due to the fact that some the symptoms of yaws can be similar to those of other conditions making it difficult to differentiate between them based on clinical presentation alone. Despite the absence of cases detected in these survey the reemergence of Yaws in Nigeria remains possible, especially as cases continue to be identified in neighboring countries. Other countries where eradication program took place decades ago such as Liberia, Malaysia and Philippines have also recently identified lately [13,21,22]. In Malaysia, yaws was presumably eliminated during the 1960s, with the last reported case published in 1985 [21]. However, in a 2021 publication, Malaysia identified and confirmed a case from the ‘Batek’ Tribe after decades of presumed elimination in the country, this case had already progressed to secondary stage before it was identified and treated due to misdiagnosis. This suggests that yaws could still be present in a region marked free for years.

### Limitation of study

In this study, the authors considered purposive selection of only the wards with hard-to-reach communities in the previously reported hyperendemic LGAs in Old Nsukka Division. Although efforts were made to intensify yaws awareness campaign in the contiguous wards, this selection may have missed yaws cases unreported to the field workers. In addition, our study was conducted only in one region in southeast Nigeria. However the findings of this study remain valuable because the study location previously reported very high numbers of yaws cases in the 1950s. Moreover, this study is considered relevant because yaws is known to be endemic in remote, hard-to-reach communities which were prioritized in this large scale survey.

### Conclusion and recommendations

Whilst no confirmed case of yaws was detected during this study we recommend that more studies be conducted in other parts of the country to help answer the open question, “Is there still yaws in Nigeria?”. Arguably, the findings of this study may not accurately determine whether or not yaws still exist in Nigeria, given the fact that the study was conducted only in a particular region in Nigeria. There is therefore need for healthcare workers across Nigeria to be trained to identify the presentation and standard management of yaws, especially health workers who live or work in rural, remote, locations often referred to as ‘end-of-the-road’ communities. Furthermore, Massive yaws campaigns and continued sensitization efforts in at-risk communities are also required to help the country establish if yaws is still present. These activities can be integrated into other skin NTDs and programmes with large population coverage such as immunization to ensure sustained surveillance.

## Supporting information

S1 Data Supporting Data. (XLSX)
